# Dynamic Deflection of a Railroad Sleeper from the Coupled Measurements of Acceleration and Strain

**DOI:** 10.3390/s18072182

**Published:** 2018-07-06

**Authors:** Sung-Ho Joh, Katherine Magno, Sung Ho Hwang

**Affiliations:** 1 Department of Civil and Environmental Engineering, Chung-Ang University, Seoul 06974, Korea; engr.kmagno@gmail.com; 2 Advanced Railroad Civil Engineering Division, Korean Research Railroad Institute, Gyeonggi-do 16105, Korea; forever7@krri.re.kr

**Keywords:** deflection, railroad sleeper, structural health monitoring, ballast track, computer vision

## Abstract

Dynamic deflection of a railroad sleeper works as an indicator of ballast stiffness, reflecting the health conditions of a ballast track. However, difficulty exists in measuring dynamic deflection of a railroad sleeper by conventional deflection transducers such as a linear variable differential transformer (LVDT) or a potentiometer. This is because a fixed reference point is unattainable due to ground vibrations during train passage. In this paper, a patented signal processing technique for evaluation of pseudo-deflection is presented to recover dynamic deflection of a railroad sleeper using a coupled measurement of acceleration and strain at the concrete sleeper. The presented technique combines high-frequency deflections calculated from double integration of acceleration and low-frequency deflections determined from strains. Validity of the combined deflections was shown by the deflections measured with a camera target on a concrete sleeper, captured by a high-resolution DSLR camera with superb video capturing features and processed by computer vision techniques, such as Canny edge detection and Blob analysis.

## 1. Introduction

Dynamic deflection of a railroad sleeper is a good indicator of the health condition of a ballast track. Deflection at a sleeper is an output of a track system in response to a given train loading. Therefore, dynamic deflections of a railroad sleeper have been measured for more than several decades for various projects. These include the development of an environmentally-friendly concrete sleepers [1], stiffness evaluation of a ballast track for a high-speed railway, and stability checks of an approach block between a bridge and a soil embankment.

Conventionally, practitioners in Korea have used a linear variable differential transformer (LVDT) or a potentiometer to carry out the deflection measurement of a sleeper. The apparatus was mounted on a short steel rod driven into ballast in the vicinity of a track. The sleeper deflections measured by LVDT’s or potentiometers tend to be underestimated due to movement of a mounting rod induced by train loading. Particle velocities of a sleeper, measured by 1- or 2-Hz geophones, have also been introduced to evaluate deflections through numerical integration. Deficit frequency components (typically 0–7 Hz) in the geophone-based signals lead to modification of the signals by low-pass and high-pass filters [1,2]. Even after filtering, the resulting deflections tend to be upwardly shifted, therefore using peak-to-peak deflections rather than just maximum deflections is recommended [3,4]. Recently, however, some successful techniques [5,6,7,8] have been reported to reproduce deflections reliably from accelerations or velocities of railroad tracks, ground surface, and other structural members. Other effective approaches of measuring sleeper deflections include piecewise integration of an acceleration history [9], and an LVDT mounted on the settlement pegs embedded into ballasts [10].

Additionally, the computer vision technique is currently a popular method of capturing digital images of deflected sleepers during train passage, digitized by a variety of algorithms such as 2-D image correlation, Canny edge detection [11,12], Blob analysis [13] and others [14]. Visual identification of sleeper deflections from video images is attractive, and several successful cases have been reported to yield reasonably accurate results [15]. When capturing images of sleeper deflections, the camera should be placed at a distance from the track to avoid shaking of the camera body itself, which occurs due to ground vibration and air turbulence during train passage.

Review of the available methodologies for the measurement of dynamic sleeper deflections reveals that there remains a demand for an accurate, reliable and practical method of measuring dynamic sleeper deflections. Rather than highly sophisticated equipment, such as an optical laser device and a professional video camera, the use of conventional instrumentation sensors adopted for a routine structural integrity assessment, such as accelerometers, strain gauges, etc., is preferable. The demand is more crucial if dynamic sleeper deflections must be monitored continuously for an extended period, such as a month or a year. In this paper, a stable and robust solution for the deflection measurement of a concrete sleeper is discussed. The solution is based on a patented signal processing technique for infrastructures named, “Evaluation of Pseudo-Deflection Method (PDM)”. The discussion includes the theoretical background, an algorithm for evaluating sleeper deflections, and a hardware suggestion for dynamic data acquisition.

The validity of sleeper deflections from the PDM was demonstrated by comparison with the deflections determined from video images of a camera target on the sleeper. Video images of a camera target were captured during train passage by a DSLR camera with a 600-mm zoom lens, 4 k resolution and a 120-frames-per-second capability. The individual image frames in the video were processed using two different computer vision (CV) techniques to determine the dynamic deflections of a sleeper. The CV techniques adopted for this validity test were Canny edge detection and Blob analysis.

## 2. PDM for a Concrete Sleeper

**Figure 1:**
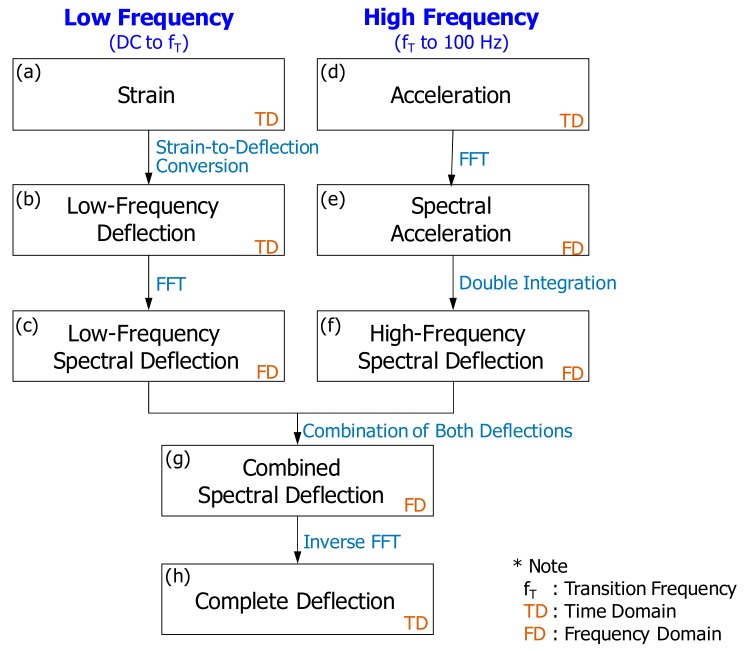
Overview of the evaluation of Pseudo-Deflection Method (PDM).

In the PDM, both acceleration and flexural strain are simultaneously measured at the same sleeper. Results from the simultaneous measurement indicate that flexural strain is synchronized with acceleration during data acquisition. An accelerometer and a strain gauge should be installed in close proximity, so that both the measured acceleration and flexural strain reflect the dynamic response of a specific point on the sleeper. The accelerations measured in the time domain (Figure 1a) are transformed to spectral accelerations in the frequency domain (Figure 1b). Numerically, deflection can be determined from the double integration of acceleration. However, there is a practical problem with the integration. When an acceleration in the frequency domain (or spectral deflection) is expressed as shown in Equation (1), the integration to determine spectral deflection is performed by Equation (2).

(1) $$\ddot{U}(\omega )={\ddot{U}}_{\omega }e^{-i\omega t}$$

(2) $$U(\omega )=\frac{1}{{\left(-i\omega \right)}^{2}}\ddot{U}(\omega )$$

where U(ω) and $\ddot{U}(\omega )$ are spectral deflection and acceleration in an exponential form, ${\ddot{U}}_{\omega }$ is acceleration at the circular frequency of ω, and t is time. The problem in the integration arises because most accelerometers are not sensitive to low frequency vibrations (e.g., vibrations lower than 3–4 Hz in the case of a PCB 353B68 accelerometer). Therefore, acceleration information over these frequencies (0 to 3–4 Hz) is not available in the data measured by accelerometers. As a result, the integration based on Equation (2) produces a strange deflection shape due to the lack of low-frequency information.

In this situation, the PDM provides the right solution to this problem for accelerometers, by borrowing low-frequency deformation from strains measured at the same location for accelerometers. Strain gauges are typically sensitive and display reliable performance up to 20 Hz vibrations. Therefore, the strain gauge is an attractive alternative sensor to measure low-frequency information of sleeper deflections. However, strain should first be converted to deflection before combination with the deflections from the double-integration of acceleration. The conversion of strain to deflection is made by exploiting the predefined relationships between strain and deflection at a specific point of a sleeper. The relationships should form a structure-specific equation and, in this research, were derived separately for a concrete sleeper. In this paper, the relationships between strain and deflection for a concrete sleeper were derived using the finite element analysis, discussed in the next section.

One of the practical strategies taken in the PDM is to obtain a deflection shape (or a non-scaled deflection curve) from the strain-to-deflection conversion. The resulting non-scaled deflection curve is later scaled to the final deflection curve by matching the strain-converted deflection with the acceleration-derived deflections at individual frequencies. In performing the PDM, a non-scaled deflection curve is preferred. This is because geometrical information and material properties of concrete sleepers are not required in determining a non-scaled deformation curve. This is described in detail in Section 3 and Section 4.

In summary, the PDM utilizes both a strain gauge and an accelerometer. The strain gauge provides low-frequency information related to a general deflection shape, and the accelerometer renders higher frequency information to determine absolute magnitudes of the deflections. Both low- and higher-frequency information on deflection constructs a complete deflection history of a concrete sleeper.

## 3. Relationships between Strain and Deflection of a Sleeper

Low-frequency deflections (e.g., less than 3–4 Hz) form the overall shape of sleeper deflections induced by train passage. Most dynamic sensors such as geophones and accelerometers (not including seismometers) do not provide reliable information in this low frequency range. The PDM introduced a strain gauge to obtain the low-frequency deflections induced during train passage of a sleeper. However, strains measured by a strain gauge are not compatible with the deflections in most infrastructures. Therefore, a theoretical tool to convert strain to deflection, specific to a concrete sleeper, is needed.

**Figure 2:**
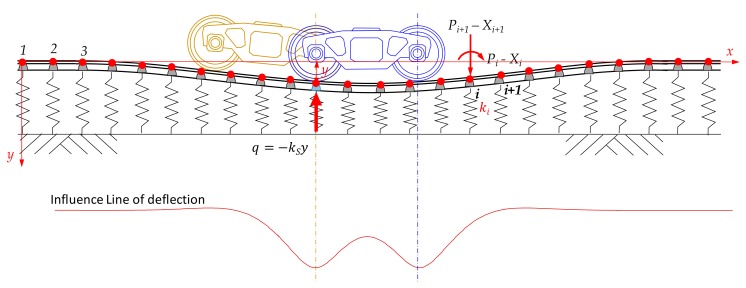
Influence line at a sleeper during train passage.

Firstly, the transient load applied by a train bogie to a sleeper is investigated, as shown in Figure 2. The ballast is modeled with a series of springs, which deform in proportion to the individual load delivered by a bogie. This spring-based model of a geotechnical material is called Winkler foundation (Equation (3)), and its solution was released in 1867 [16]. The basic differential equation for the Winkler model is expressed as Equation (3), where *y* is vertical displacement of a rail, q is contact pressure and $k_{S}$ is the subgrade reaction coefficient.

(3) $$EI\frac{d^{4}y}{dx^{4}}=q=-k_{S}^{}y$$

Bowles [16] proposed a finite element solution for the Winkler model. In the model, the rail was modeled as a beam element, and the ballast was modeled using a soil spring with soil reaction coefficient, $k_{S}$. The boundary conditions at both ends of the rail are free. Three fundamental equations in the finite element analysis are the relationships between external nodal force (**P**) and internal member force, the relationships between external nodal displacements (**X**) and internal member deformation, and the relationships between internal member forces and internal member displacements. These equations are utilized to determine external nodal displacements as shown in Equation (4). In the equation, matrix **A** is a bridging constant between external nodal forces and internal member forces, and matrix **S** is a stiffness matrix.

(4) $$X={\left(ASA^{T}\right)}^{-1}P$$

(5) $$P=ASA^{T}X$$

Equations (4) and (5) were implemented using MATLAB functions to compute nodal deflections (and rotations) and forces (and moments). Utilizing Equation (4), calculations of displacements were repeated for a moving load, which lead to the influence line of the rail deflection, as shown in Figure 2. As the influence line of deflection indicates the deflection developed at a sleeper when a loading position is shifted from one point to the other, the influence line is the same as the change of deflections during train passage, that is, a time-history of deflections at the sleeper.

**Figure 3:**
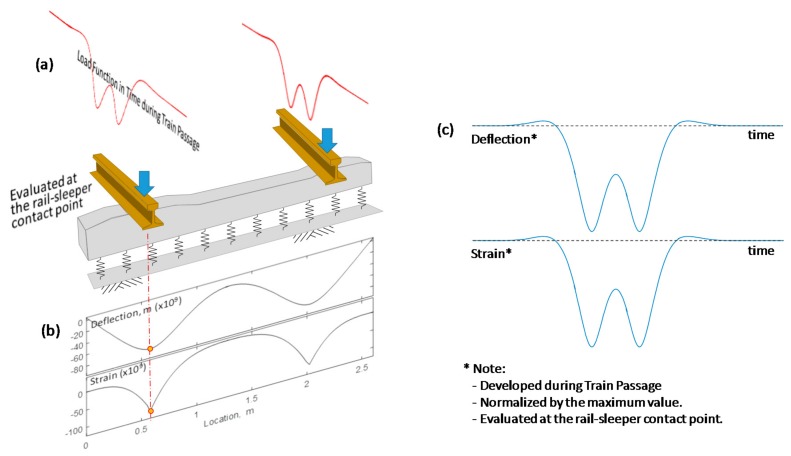
Relationships between strain and deflection: (**a**) load function in time during train passage; (**b**) deflection and strain functions along the sleeper; (**c**) time-histories of deflection and strain developed during train passage.

A pair of a transient loads, proportional to the deflection history shown in Figure 2, were applied to an individual concrete sleeper (Figure 3a). The analytical solutions proposed by Bowles [16] were employed to calculate deflections and moments (M) at the top surface of a sleeper for a pair of static loads. The moments were converted to strains (ε), parallel to the longitudinal direction of a sleeper, using the relationships in Equation (6), where z is the distance from the neutral axis to the top surface of the sleeper, E is Young’s modulus and I is the moment of inertia.

(6) $$\varepsilon =\frac{\sigma }{E}=\frac{Mz}{IE}$$

The resulting deflections and strains are shown in Figure 3b, which are the results for a single static load in the overall loading history. With the linear-elastic assumption for the trackbed system, a complete response of a sleeper can be calculated for the entire loading history, using the finite element method for a Winkler foundation. The resulting deflection and strain histories are shown in Figure 3c. When installing a strain gauge, the most practically feasible location for a strain gauge is as close as possible to the rail-sleeper contact point. Even though the strain gauge is not located directly beneath the rail–sleeper contact, the general shape of a strain history remains the same as the one in Figure 3c.

The discussion in this section is based on the fundamental assumption that ballast has a uniform stiffness. With a different ballast condition like a hanging sleeper, the results may not be simplified like those in this section. In a typical situation, however, the deflection of a concrete sleeper has the same shape as the strain measured at the top surface of a concrete sleeper, as shown in Figure 3c. Therefore, the strain-to-deflection conversion in the case of a concrete sleeper does not require any particular formula, and it is rational to take a strain history as a deflection history for the PDM.

## 4. Field Measurements of Sleeper Deflections during Train Passage

**Figure 4:**
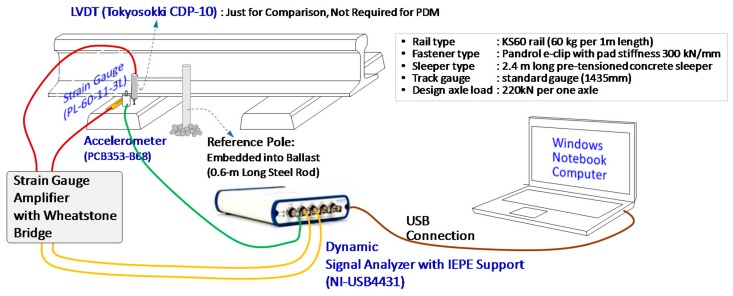
Equipment for the PDM.

Application of the PDM was made to a real concrete sleeper, as shown in Figure 4. At the top surface of a concrete sleeper, three sensors, including an accelerometer (PCB-353B68 by PCB Piezotronics, New York, NY, USA), strain gauge (PL-60-11-3L by Tokyo Sokki, Tokyo, Japan), and LVDT (Tokyo Sokki, CDP-10) were installed. The LVDT was employed for just the comparison between PDM deflections and LVDT deflections, and not used for PDM. The accelerometer, PCB353-B68, has a sensitivity of 100 mV/g, a frequency range of 1 to 10,000 Hz and a measurement range up to 50 g pk. The strain gauge was adopted to measure bending strain parallel to the longitudinal direction of a sleeper. The model of a strain gauge used for this research was Tokyo Sokki PL-60-11-3L with a resistance of 120 Ohm, a gauge length of 60 mm. The LVDT, Tokyo Sokki, CDP-10, is a displacement transducer, good for both static and dynamic measurements, and has a capacity of 5 mm, a sensitivity of 2000 micro strain/mm and a rated output of 5 mV/V.

### 4.1. Sleeper Deflections Determined by the PDM

**Figure 5:**
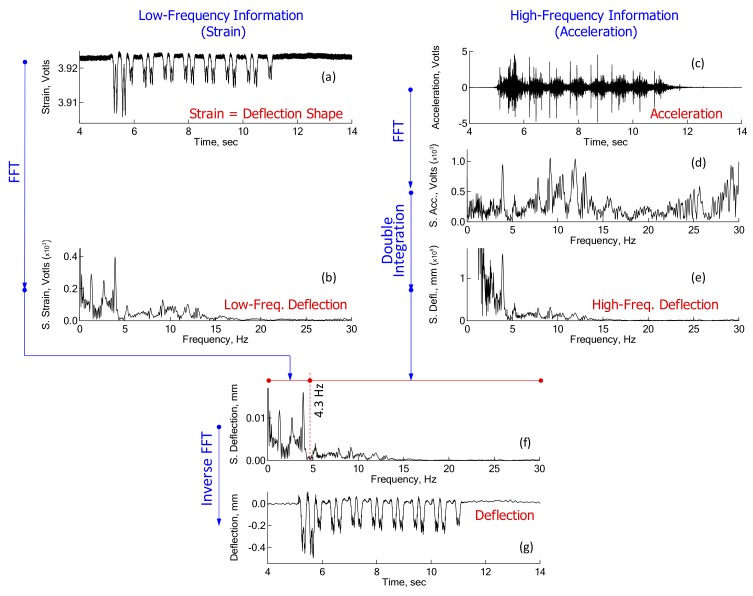
Illustration of the PDM applied to a passenger train: (**a**) Measure strain history, (**b**) Strain spectrum from FFT, (**c**) Measures acceleration history, (**d**) Acceleration spectrum from FFT, (**e**) Deflection spectrum from double integration of acceleration, (**f**) Combined deflection spectrum, and (**d**) Deflection history from inverse FFT of combined deflection spectrum.

Demonstration of the PDM for estimation of sleeper deflections was made with real measurements, as shown in Figure 5. In this figure, the stepwise procedure of Figure 1 was revisited, using the real data to gain a better understanding. In the middle of the procedure, calibration of acceleration signals was made during double integration, as shown in Figure 5e. One of the key parameters in the PDM is transition frequency. The transition frequency between low- and high-frequency deflection is different from case to case; for the case of a concrete sleeper on a soil trackbed, it typically ranges from 4 to 7 Hz. Transition frequency is defined as the lowest frequency in the acceleration-converted deflection spectrum, which displays the same shape as the strain-converted deflection spectrum. In the case of Figure 5, the transition frequency is 4.3 Hz. Hence, the spectral deflections from 4.3 Hz and above (for say 10 Hz or so) in the acceleration-converted deflection spectrum show the same trend as those in the strain-converted deflection spectrum.

The resulting deflections of the sleeper look similar in shape to the strains. However, as all the high-frequency noises were filtered out from the strains, the final deflections show a clean and crisp shape, which is sufficient to investigate the response due to bogie loads. The two large, initial deflections, corresponding to an engine train, are also worth noting.

**Figure 6:**
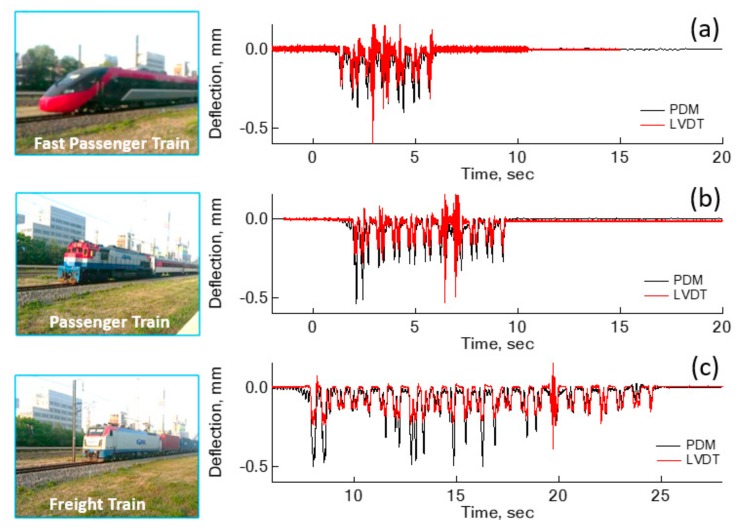
Further examples of deflections evaluated by the PDM, compared with linear variable differential transformer (LVDT) deflections: (**a**) ITX-Saemaeul Train, (**b**) Moogunghwa Train, and (**c**) Freight Train.

Additional sleeper deflections of a concrete sleeper were estimated for three different types of trains, as shown in Figure 6. The ITX-Saemaeul in Figure 6a is a fast passenger train with six carriages, which runs at an average velocity of 120–130 km/h. Moogunghwa, in Figure 6b, has nine carriages for passengers, and its engine train is heavier than other passenger carriages. Its average velocity is 100–110 km/h. The freight train in Figure 6c hauls 22 carriages with some fully loaded, additionally, the engine train is heavier than other carriages.

An ITX-Saemaeul train, a fast passenger train shown in Figure 6a, has a light engine train, compatible with the weight of a passenger carriage, observed from the peak deflections of bogies at each carriage. The largest peak deflection was estimated to be 0.42 mm at one of the passenger carriages, as opposed to an engine bogie. The engine train of the ITX-Saemaeul is driven by an electric motor, not a diesel engine. The transition frequency between low- and high-frequency deflection was 6.0 Hz. Moogunghwa, shown in Figure 6b, another passenger train, was estimated to have the maximum deflection of 0.54 mm at the bogie of an engine train. Deflections at other passenger carriages hauled by the diesel engine ranged from 0.22 to 0.30 mm, indicating the passenger carriages are in approximately the same weight range. The transition frequency was also 6.0 Hz for this passenger train. Deflections evaluated for a freight train are shown in Figure 6c. Two peaks in the beginning of the deflection history are clear and correspond to the maximum deflections, 0.54 mm. The transition frequency adopted for the PDM is 6.5 Hz, higher than other types of trains.

Estimation of sleeper deflections were repeated for four different sleepers, located within a 200 m radius of each other, for a period of eight months. The types of trains used for the estimation of PDM deflections are those shown in Figure 6. Altogether, a total of 55 PDM measurements were performed in order to estimate the deflections at concrete sleepers. Based on these measurements for the PDM, it was found that the transition frequency ranges from 4 to 7 Hz, regardless of running speed and weight of the train. Therefore, it is reasonable to set a general guideline for the transition frequency of 7 Hz, the highest frequency in a preferred frequency range, as more low frequency components lead to a better deflection shape.

Additional experimental observations are required to set up a general guideline for the transition frequency, generalized for different types of sleepers and ballast conditions. Once the general guideline for a transition frequency is found, the analysis procedure using the PDM can be fully automated and utilized for monitoring programs of sleepers in unstable conditions, such as embankments on soft clay, approach blocks and others.

### 4.2. Underestimation of Sleeper Deflections Measured by an LVDT

An LVDT has been the conventional sensor for the measurement of sleeper deflections during train passage in Korea for the past several decades. A reference pole to mount an LVDT is installed approximately 0.5–0.7 m deep in the ballast track close to the sleeper. Ground vibration induced by a running train vibrates the reference pole, leading to underestimation of sleeper deflections.

Comparisons between deflections estimated by the PDM and measured by LVDT were made in Figure 6. In general, LVDT deflections are smaller than PDM deflections. In the case of an engine carriage of the freight train, LVDT deflection is as small as 43% of PDM deflections, at 0.28 mm. However, the difference becomes less for a light carriage like an unloaded freight carriage. Therefore, the reference steel rod to mount the LVDT has a greater downward movement with the heavier carriage. The reliability of LVDT deflections for a diesel engine car specifically, is too low to use in real practice.

## 5. Laboratory Tests for Reliability of PDM

**Figure 7:**
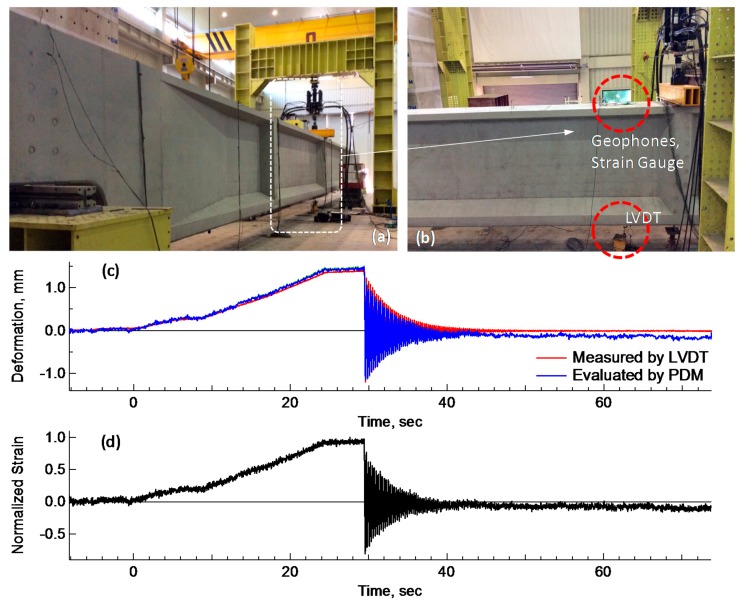
Comparison of PDM deflections and LVDT deflection in a laboratory test: (**a**,**b**) lab tests for deflection measurements; (**c**) comparison of PDM and LVDT Deflections; (**d**) strain history.

A more intensive laboratory test was made on the reliability and accuracy of the PDM for estimation of deflections. In the laboratory test (Figure 7a), a concrete beam was used due to low-frequency behavior and availability of the model. In the test, a pair of geophones and a strain gauge were installed at the top surface of a concrete beam, shown in Figure 7b. The aim of the test was to investigate the structural characteristics of free vibration, where accelerations and deflections of the beam were measured directly by an LVDT installed at the bottom of the same beam and indirectly by the PDM. The PDM deflections were compared with the LVDT deflections. In this PDM test, a 2.0 Hz geophone was used instead of an accelerometer, to enable better sensitivity for low frequency measurements.

A comparison of the resulting deflections was provided in Figure 7c. The deflection history has two parts: a slowly increasing deflection, and a free-vibration deflection. The resonant frequency for the free vibration was selected as 4.27 Hz, even though any frequency in the range from 3.0 to 7.0 Hz was suitably working as the transition frequency. The PDM deflections are in good agreement with the LVDT deflections. However, there is a general tendency for the difference between two deflections to increase with time. Deflections have an initial difference of 0.067 mm between PDM and LVDT deflections, 0.08 mm at 29.4 s, the initial point of free vibration, and 0.3 mm at 73.3 s. The decreasing trend of the PDM deflections is identical to the trend of strain history shown in Figure 7d. A strain gauge shows a drift phenomenon in the measurements, which was a typical observation made in both the lab tests and field tests for sleepers. Thus, the drift in the PDM deflection history is attributed to the strain sensor, not the PDM itself. The laboratory test revealed that the PDM is reliable for estimating deflections within a limited accuracy, which is controlled by performance of a strain gauge, not the PDM itself.

## 6. Validity of the PDM Investigated by Computer Vision

Feasibility of the PDM for the sleeper deflections during train passage was confirmed through multiple measurements under various conditions, such as different types of trains, geological sites, running speeds and others. Now, the validity of deflections measured by the PDM is investigated using the computer vision technique in this section.

**Figure 8:**
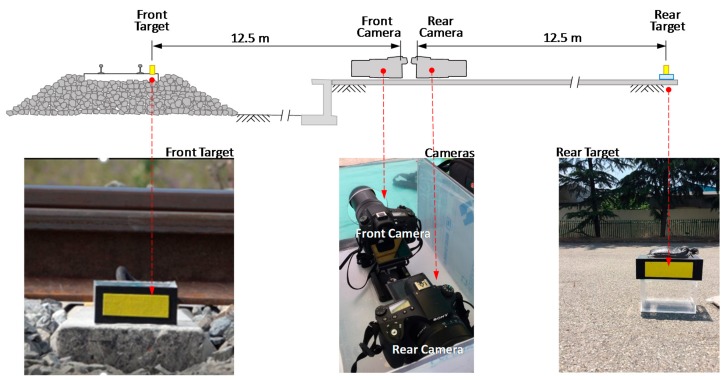
Setup for capturing video images of sleeper vibration during train passage.

As shown in Figure 8, together with the sensors for the PDM, two DSLR cameras (RX 10 Mark 3 by SONY, Tokyo, Japan) were installed 12.5 m from the railroad track to capture the movements of two yellow rectangular targets, one at the same sleeper and the other 25 m away from the railroad track in the array perpendicular to the track, as shown in Figure 8. To digitize the captured target images, a Canny edge detection technique [11,12], was employed. Later, the deflections digitized by Canny edge detection technique were compared with the results by Blob analysis [13] for validation. Canny edge detection technique extracts edges by: (1) applying a Gaussian filter which smooths the image; (2) evaluating gradients to identify color change; and (3) adopting a threshold for the characterization of edges. Blob analysis analyzes pixels of images in the format of a blob, which is a rectangle with homogeneous area. In the Blob analysis, the images are first smoothed by a Gaussian filter, then treated by a color threshold to detect blobs in a rectangular area consisting of pixels with constant contrast.

**Figure 9:**
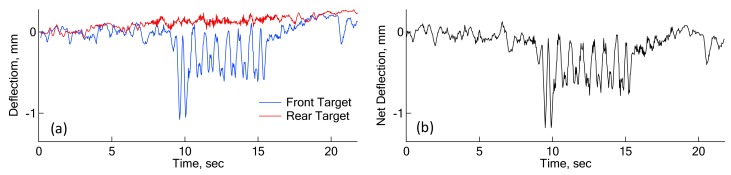
Deflections determined from Canny edge detection technique: (**a**) deflections of front and rear targets; (**b**) net deflections between front and rear targets.

In Figure 8, a rear camera was employed in the measurement of dynamic sleeper deflections to remove vibrations of the camera itself caused by ground vibration induced by a running train. The rear target is 25 m away from the railroad track, and should be stationary even during train passage. Therefore, any vibrations shown by the rear target could be related with the ground vibrations at the place where the camera was mounted. Figure 9 shows a pair of deflections from front and rear targets. Deflections from a rear target are not sufficient to influence the deflections of the front target. This indicates the camera-mounted ground is not shaken significantly by a running train, and the correction using the rear target may not be required. In fact, the net deflections in Figure 9b are practically the same as the front deflections in Figure 9a.

**Figure 10:**
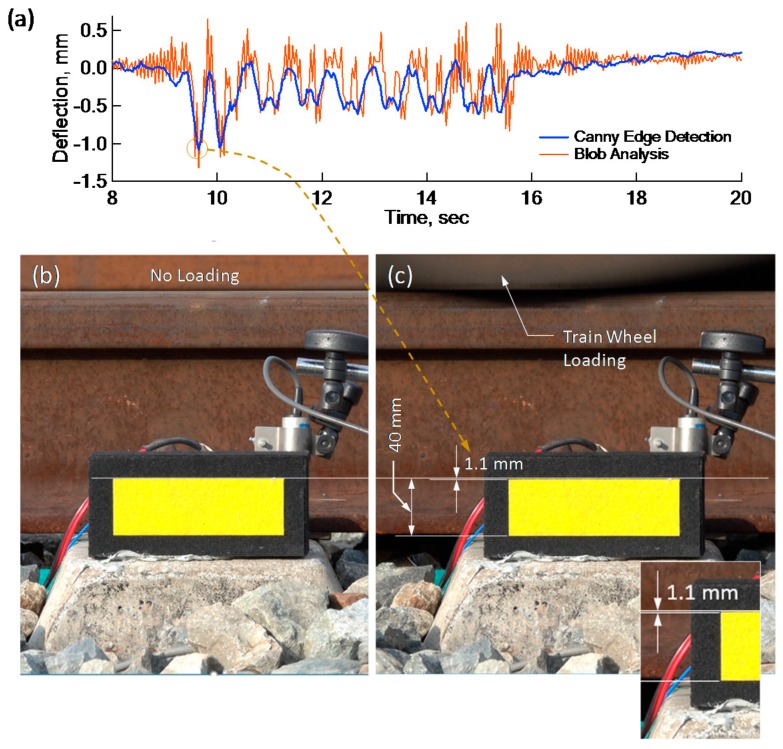
Validity of Canny edge detection technique: (**a**) Comparison of Canny edge technique and Blob analysis, (**b**) Target image on unloaded sleeper, and (**c**) Target image on loaded sleeper.

**Figure 11:**
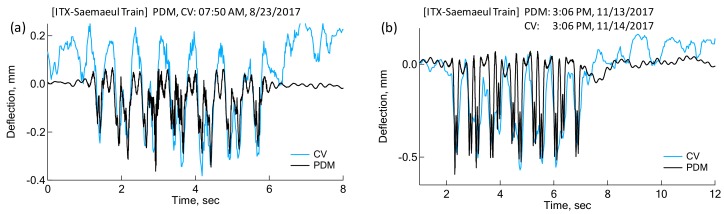
Deflections evaluated by PDM and computer vision technique: (**a**) measured at the same time; (**b**) measured at a different day for the same train.

The results from the Canny edge detection technique were compared with those from Blob analysis, another computer vision technique. Figure 10a shows a good match in peak deflections and a general deflection shape. The peak deflection was calculated directly from the stationary image with a train wheel loaded right on the sleeper, shown in Figure 10b,c. Based on the height of the yellow marker, the peak deflection was evaluated to be 1.1 mm, which is compatible with that which was calculated by both computer vision techniques. Finally, comparisons between deflections by the PDM and deflections by the Canny edge detection technique were made in Figure 11. In Figure 11a, CV and PDM deflections were evaluated at the same time for the same train. The downward deflections of PDM due to wheel loading are comparative to CV deflections. However, upward CV deflections were highly fluctuating both before and after the passage of the train. This could probably be attributed to a strong wind blow or some other causes at the time of capturing, leading to camera vibrations.

Therefore, if only the downward deflections are considered for comparison, averages of peak deflections by PDM and CV technique are 0.224 mm and 0.265 mm, respectively. The maximum peak deflections are 0.363 mm and 0.382 mm for PDM and CV techniques, respectively. The difference between two techniques in the average and peak deflections is 0.15 mm (15% of the peak CV Deflection) and 0.05 mm (5% of the peak CV Deflection). In the case of Figure 11b, the measurements for PDM and CV techniques were performed at a different time due to weather conditions and field situations. Video capturing of sleeper deflections was carried out one day after the measurements for PDM were carried out. The train was the same one running at the same time of the day, with only the number of passengers on the train potentially differing. Therefore, both test conditions could be considered as identical in ballast condition, sleeper, train type, train weight, train speed, etc. Interestingly, Figure 11b shows a good agreement in peak deflections and shape between the two methods. The CV deflections in Figure 11b do not show fluctuations as significant as those in Figure 11a. However, there are some fluctuations in CV deflections after the train has passed. The average of peaks in the deflection history are 0.47 mm and 0.46 mm, respectively for PDM and CV deflections. In addition, the maxima of peaks for the PDM and CV technique are 0.59 mm and 0.57 mm, respectively. The difference is only 0.01 mm and 0.02 mm for average and maximum deflections, which corresponds to 2% and 3% of the peak deflections. Hopefully, in the future a more accurate and better comparison between CV and PDM deflections can be made using a professional video camera with a high zoom lens and a sophisticated CV technique.

## 7. Summary and Conclusions

Dynamic sleeper deflection works as an indicator for the health condition of a ballast track. A practical, reliable and affordable technique for the measurement of sleeper deflections has been presented. The new method called the PDM makes coupled measurements of acceleration and flexural strain. It combines low-frequency deflections of strains and high-frequency deflections of accelerations. Results and findings obtained during the research for the PDM are summarized as follows:

- Validity of the PDM was shown by two different computer vision techniques (Canny edge detection technique and Blob analysis) and by visual inspection of a stationary digital image of a sleeper deflected directly under a running train wheel.
- Accuracy and reliability of the PDM depends on the performance of the strain gauge, based on the laboratory tests comparing LVDT deflections and PDM deflections.
- Transition frequency between low-frequency and high-frequency deformations ranges from 4 to 7 Hz in the case of the ballast tracks at a geotechnical site studied in this research. However, a ballast track on a rigid bridge or a tunnel inverter may require a different transition frequency and therefore must be investigated for a suitable selection of transition frequency.
- Comparison between PDM deflections and LVDT deflections indicates that LVDT underestimates the deflections, specifically for a heavy diesel engine train. If LVDT is incorporated for deflection measurements, a reference rod for an LVDT should be installed as far away as possible from the rail. Embedding a reference rod near a sleeper, as practiced in Korea, should be avoided.

## 8. Patents

The PDM is patented in Korea (Registration number is 10-1520231). Title of the patent is “Technique to determine deflection of infrastructures based on grafting of acceleration and strain time histories.”
